# Long-term effects of an acute and systemic administration of LPS on adult neurogenesis and spatial memory

**DOI:** 10.3389/fnins.2014.00083

**Published:** 2014-04-21

**Authors:** Jorge Valero, Giorgia Mastrella, Ismael Neiva, Silvia Sánchez, João O. Malva

**Affiliations:** ^1^Neuroprotection and Neurogenesis in Brain Repair Group, Center for Neuroscience and Cell Biology, University of CoimbraCoimbra, Portugal; ^2^Institute for Interdisciplinary Research, University of CoimbraCoimbra, Portugal; ^3^International Master Degree in Neuroscience, Department of Life Sciences, “Università degli Studi di Trieste”Trieste, Italy; ^4^Faculty of Medicine, Institute for Biomedical Imaging and Life Sciences, University of CoimbraCoimbra, Portugal

**Keywords:** 3xTg-AD mouse, doublecortin cells, dentate gyrus, cognitive reserve, hippocampus, inflammation, microglia, synaptic puncta

## Abstract

The cognitive reserve is the capacity of the brain to maintain normal performance while exposed to insults or ageing. Increasing evidences point to a role for the interaction between inflammatory conditions and cognitive reserve status during Alzheimer's disease (AD) progression. The production of new neurons along adult life can be considered as one of the components of the cognitive reserve. Interestingly, adult neurogenesis is decreased in mouse models of AD and following inflammatory processes. The aim of this work is to reveal the long-term impact of a systemic inflammatory event on memory and adult neurogenesis in wild type (WT) and triple transgenic mouse model of AD (3xTg-AD). Four month-old mice were intraperitoneally injected once with saline or lipopolysaccharide (LPS) and their performance on spatial memory analyzed with the Morris water maze (MWM) test 7 weeks later. Our data showed that a single intraperitoneal injection with LPS has a long-term impact in the production of hippocampal neurons. Consistently, LPS-treated WT mice showed less doublecortin-positive neurons, less synaptic contacts in newborn neurons, and decreased dendritic volume and complexity. These surprising observations were accompanied with memory deficits. 3xTg-AD mice showed a decrease in new neurons in the dentate gyrus compatible with, although exacerbated, the pattern observed in WT LPS-treated mice. In 3xTg-AD mice, LPS injection did not significantly affected the production of new neurons but reduced their number of synaptic puncta and impaired memory performance, when compared to the observations made in saline-treated 3xTg-AD mice. These data indicate that LPS treatment induces a long-term impairment on hippocampal neurogenesis and memory. Our results show that acute neuroinflammatory events influence the production of new hippocampal neurons, affecting the cognitive reserve and leading to the development of memory deficits associated to AD pathology.

## Introduction

Newborn excitatory granule cells are continuously generated from precursor cells located in the subgranular zone (SGZ) of the adult dentate gyrus (DG). These newly generated cells integrate into pre-exiting hippocampal memory circuits playing a role in the codification of spatial memory (Altman and Das, 1965; Dupret et al., 2007; Deng et al., 2009; Garthe et al., 2009). The continuous addition of new neurons to the DG is a key component of the hippocampal neurogenic reserve (Kempermann, 2008), and a structural element of the cognitive reserve (Nithianantharajah and Hannan, 2009). The term cognitive reserve have acquired great relevance in the field of neurodegenerative diseases with a special impact in Alzheimer's disease (AD). Thus, a great interest exists to understand how life-style related factors favor or impede the development of a healthy cognitive reserve, affecting cognitive function.

AD is a neurodegenerative disorder characterized by cognitive decline accompanied by the accumulation of Aβ plaques, neurofibrillary tangles, neuroinflammation and neuronal loss (Querfurth and LaFerla, 2010). For many years, the accumulation of Aβ peptides in the brain has been considered the major trigger of AD pathology (Hardy and Selkoe, 2002; Hardy, 2009). However, several studies failed to find a correlation between amyloid deposition levels and loss of cognitive function, suggesting that elevated concentrations of Aβ peptide in the brain are necessary but not sufficient to induce AD pathology (Pike et al., 2007; Jack et al., 2009; Rowe et al., 2010). Therefore, new AD hypothesis have emerged to conciliate these phenomena. Recently, the involvement of the brain cognitive reserve in the development of this pathology has gained a renewed interest (Stern, 2012; Ewers et al., 2013; Lo et al., 2013). Furthermore, systemic inflammation may induce neuroinflammation that impairs some key elements of the cognitive reserve (neurogenesis or synaptic plasticity) and has been directly related to AD and even suggested to trigger this pathology (Herrup, 2010).

LPS-mediated systemic inflammation triggers a cascade of cellular and molecular events that are able to initiate neuroinflammation (Godbout et al., 2005; Qin et al., 2007; Dantzer, 2009), affect cognitive function (Pugh et al., 1998; Arai et al., 2001; Sparkman et al., 2005, 2006; Lee et al., 2008; Dobrin et al., 2010; Ormerod et al., 2013) and reduce adult neurogenesis (Monje et al., 2003; Bastos et al., 2008; Bachstetter et al., 2010; Fujioka and Akema, 2010; Sierra et al., 2010). However, the long-term effect of acute systemic inflammation on hippocampal neurogenesis and memory function in wild type (WT) or mouse models of AD has not been properly explored.

In this work, we examined the effect of an acute intraperitoneal injection of LPS in WT and triple transgenic mouse model of AD (3xTg-AD) at early pathological stages. We found that this single LPS administration has a profound long-term impact in cognition, impairing spatial memory in both WT and 3xTg-AD mice. LPS had a weaker effect on memory in WT mice but elicited an increase in the number of new microglial cells, a decrease in the number of doublecortin (Dcx)-positive new neurons and reduced the size of their dendritic tree and number of synaptic puncta. These results demonstrate that a single neuroinflammatory episode is able to induce a long-term impairment of the hippocampal neurogenic reserve and challenge the capacity of the central nervous system to maintain normal memory function.

## Materials and methods

### Ethics statement

Animal experimental procedures were performed in order to minimize the number of animals used and exposure to stress and suffering, in accordance with institutional animal house, national (Bioterio FMUC; License n°520.000.000.2006, from the Portuguese animal welfare authorities) and European Community guidelines (86/609/EEC; 2010/63/EU).

### Mice

**Figure 1:**
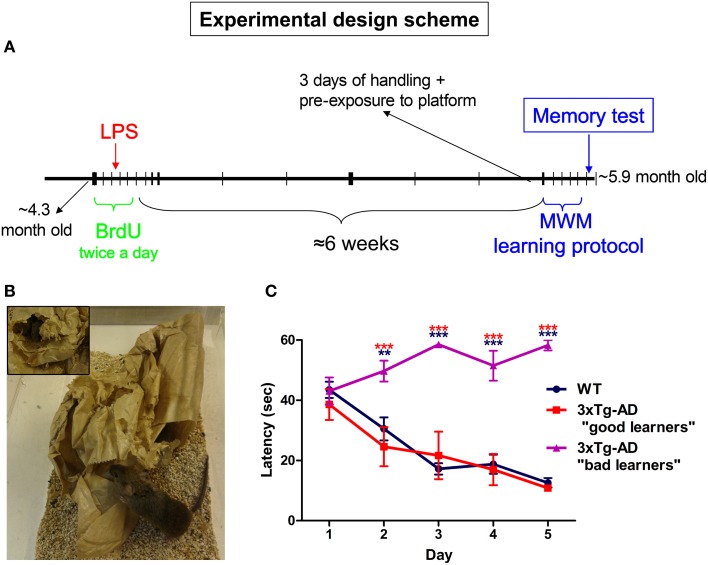
**Experimental design**. **(A)** 5-bromo-2′-deoxyuridine (BrdU) was administered (100 mg/Kg) intraperitoneally for 5 days (twice per day with 8 h interval). On day third of BrdU treatment animals received a single intraperitoneal injection of saline (PBS) or 1 mg/Kg of LPS. Six weeks after last BrdU injection all mice were submitted to MWM training and test protocol. **(B)** Mice were housed in cages containing a malleable paper bag that was changed twice a week. **(C)** Only 3xTg-AD mice which performed similarly to WT mice during MWM trial sessions were selected for this study. Data represent mean ± s.e.m. ^**^p < 0.01, ^***^p < 0.001 (red: 3xTg-AD “bad learners” vs. “good learners” mice; blue: 3xTg-AD “bad learners” vs. WT mice).

Twenty-nine WT and 26 3xTg-AD female mice were used for this study. WT mice were non transgenic/knockin mice from the same strain and genetic background as PS1 knockin mice, a hybrid 129/C57BL6. Generation of the 3xTg-AD mice has been previously described (Oddo, 2003). All mice were housed in the same room and in similar cages containing a malleable paper bag that was changed twice a week (Figure 1B). Animals were kept on a 12 h light/dark cycle and given *ad libitum* access to food and water.

### Bromodeoxyuridine and lipopolysaccharide administration

To label proliferating cells, the thymidine analog 5-bromo-2′-deoxyuridine (BrdU; B5002, Sigma-Aldrich Co. LLC) was administered (100 mg/Kg) intraperitoneally in 0.1 M phosphate buffered-saline (PBS) at pH 7.3 for 5 days (twice per day with 8 h intervals, Figure 1A). On day third after starting BrdU treatment and 4 h after the first BrdU administration, animals received a single intraperitoneal injection of saline (PBS) or 1 mg/Kg of LPS (from *Escherichia coli* 055:B5, 067K4056, Sigma-Aldrich Co. LLC) diluted in PBS (Figure 1A). Fourteen WT and 12 3xTg-AD mice were administered with saline, while 15 WT and 14 3xTg-AD mice were treated with LPS. The weight of mice was controlled from the day of LPS injection till total recovery (around 1 week later). Six weeks after last BrdU injection all mice went through the MWM training and test protocol.

### Morris water maze test

The Morris water maze (MWM) consisted of a circular pool (140 cm in diameter) containing a hidden (transparent) platform (14 cm in diameter) submerged 1 cm below water (21 ± 2°C). The pool was enclosed with black curtains, surrounded by four different black and white visual cues of 50 × 50 cm and illuminated with red light. Mice were handled during the 3 days prior to MWM training. On a pilot study we observed that 3xTg-AD mice were able to learn faster after pre-exposure to the platform. Therefore, mice from the initial MWM group were pre-exposed to the platform during the last handling session and before starting the first MWM trial. However, the morphological differences observed in newborn neurons and decreased robustness of the neurogenic niche in WT mice, compelled us to further analyze cognitive performance in WT saline-treated vs. WT LPS-treated mice. Consistently, we performed a second standard MWM protocol where animals were not pre-exposed to the platform. For each trial, mice were placed into the pool at one of four pre-defined starting points in a pseudo-random order. Animals were trained for 5 days (six trials per day) with maximum trial duration of 60 s and a minimum intertrial interval of 15 min. Mice showing thigmotaxia-like behavior (spending more than 50% of the time in a strait corridor close to the MWM pool walls) were excluded from the study. To assess memory retention, 24 h after the last trial, mice were tested in three consecutive 1 min-probe trials in the absence of the platform. All trials and probe tests were performed during the first hours of the dark/active period of the mice. Performance of the mice was recorded with a camera located at the ceiling. Latency times were collected *in situ* and test probe parameters analyzed by using the ImageJ plugin MTrackJ (Meijering et al., 2012) and wintrack software (Wolfer et al., 2001).

### Immunofluorescence staining

Ninety minutes after last MWM test mice were deeply anesthetized with 15 μ l/g of body weight of a mixture of ketamine hydrochloride (0.9 ml; Imalgene 1000, Merial) and Xylazine (0.5 ml, Rompun 2%, Bayer), and then perfused intracardially with 0.9% NaCl followed by 4% (w/v) phosphate buffered paraformaldehyde. Cerebral hemispheres were postfixed with the same fixative for 2 h, rinsed with PBS for 2 h and immersed in 30% (w/v) sucrose diluted in PBS until they sank. After cryoprotection, 40 μm-thick coronal sections were obtained with a criostat and collected in six series. Tissue sections were stored at −20°C in an antifreezing mixture made of 30% glycerol (v/v) and 30% polyethylene glycol (v/v) in 0.1 M phosphate buffer, pH 7.4.

Sections were rinsed in PBS (6 × 10 min), incubated in 1 M HCl (1 h at 37°C) to allow DNA denaturation and rinsed again in 0.1 M borate buffer (pH 8.5, 3 × 10 min) and PBS (3 × 10 min). Then, sections were immersed in blocking solution (3% Bovine Serum Albumin and 0.2% Triton X-100 in PBS) for 1 h and incubated with rat anti-BrdU (1:4000; OBT0030CX, AbD Serotec) and mouse anti-NeuN (1:4000; MAB377, Millipore), mouse anti-BrdU (1:500; clone Bu20a M0744, Dako), rabbit anti-glial fibrillary acidic protein (GFAP, 1:5000; G9269, Sigma-Aldrich Co. LLC) and rat anti-CD11b (1:3500; MCA711, AbD Serotec) in blocking solution for 48 h. For triple Dcx/PSD95/vGlut staining HCl and borate buffer incubations were omitted and tissues were incubated with goat anti-doublecortin (Dcx, 1:500; Clone C-18, SC-8066, Santa Cruz Biotechnologies), mouse anti-PSD95 (1:2000; clone K28/43, 05-494, Upstate) and rabbit anti-vGlut 1 (1:5000; 135303, Synaptic Systems) in blocking solution for 48 h. Then, tissue slices were rinsed in PBS (3 × 10 min) and immersed for 2 h in a solution containing secondary antibodies, Hoechst 33342 (1:10000; H1399, Invitrogen), 0.2% Triton X-100 and PBS. Finally, sections were rinsed with PBS (3 × 10 min) and mounted with antifading medium (Fluoroshield Mounting Medium, ab104135, Abcam). Secondary antibodies used were Alexa Fluor® 488 donkey anti-mouse IgG, Alexa Fluor® 488 donkey anti-rat IgG, Alexa Fluor® 594 donkey anti rabbit IgG, Alexa Fluor® 647 goat anti-mouse IgG and Alexa Fluor® 633 donkey anti-goat IgG (all of them at 1:1000; from Invitrogen).

### Cell number and volume quantifications

Stereological estimation of the number of BrdU and Dcx-positive cells was performed in serial sections at 240 μm rostrocaudal intervals using the optical fractionator module of Stereoinvestigator software (MicroBrightField). Briefly, contours were traced around the GCL including SGZ (12.5 μm above the GCL) by using a 20× objective. BrdU and Dcx-positive cells were counted in the entire GCL-SGZ region under a 63× oil immersion objective using a 12 μm optical dissector depth (3 μm top and bottom guard zones). Estimation of total number of double stained BrdU/NeuN and BrdU/CD11b-positive cells was performed based on the percentage of BrdU-positive cells expressing NeuN and CD11b. Hypertrophic CD11b/BrdU-positive cells (showing thick processes emerging from their cell body) were considered as reactive-like microglial cells (Figure 3C; Ziaja and Janeczko, 1999). NeuN/BrdU and CD11b/BrdU analyses were performed using confocal images obtained with a Zeiss LSM 510 Meta confocal microscope.

### Analysis of CD11b staining

Single plane confocal images were obtained from 3 septal hippocampal sections with 240 μm rostrocaudal intervals (from bregma −1.70 to −2.30 mm). An ImageJ macro was programmed to allow automatic processing of confocal images. The percentage of CD11b-stained area was quantified in eight 20 × 20 μ m regions of interest (ROIs) per image (a total of 72 ROIs were analyzed per mouse).

### Analysis of dendritic morphology

For analysis of dendritic morphology, we obtained 3D reconstructions from confocal stack images of the most mature type of Dcx-positive cells located in the septal region of the hippocampus. 3D reconstructions were performed with the Simple neurite tracer plugin (Longair et al., 2011) working on the image processing package Fiji (Schindelin et al., 2012). Reconstructed Dcx-positive dendritic trees should reach the molecular layer (ML) and branch into the GCL to be included in the study (*n* = 48 cells/group). Dendritic morphology was analyzed using 3D Sholl analysis plugin (http://fiji.sc/Sholl_Analysis) by quantification of the number of intersections between dendrites and the surface of spheres with a radius increment of 10 μm.

### Quantification of synaptic markers

The number of PSD95 and vGlut-positive puncta were analyzed in confocal stacks from the septal inner ML and outer/mid ML (Figure 5D). An ImageJ macro was programmed to allow automatic processing of confocal images concatenating the use of several image filters for: (1) background normalization (subtract background, http://imagejdocu.tudor.lu/doku.php?id=gui:process:subtract_background), (2) puncta segmentation (“Find peaks” plugin, University of Sussex, Brighton, United Kingdom: http://www.sussex.ac.uk/gdsc/intranet/microscopy/imagej/findpeaks), (3) stereological selection of puncta (Vamp3D plugin; Dumitriu et al., 2012), and (4) the use of 15 × 15 μ m automated stereological counting frames. Automatic quantification parameters were calibrated in pilot studies to match manual quantifications.

For puncta analysis in Dcx-positive dendrites, ROIs were drawn delimiting portions of Dcx-positive apical dendrites with a length of 10 ± 2 μm (*n* = 48 dendrite segments/group). Total puncta analysis was performed using confocal image stacks from the suprapyramidal inner ML and outer/mid ML regions of septal hippocampal sections (from bregma −1.70 to −2.30 mm).

### Statistical analysis

Data are expressed as means ± standard error of the mean (SEM). Statistical significance was determined using Two-Way analysis of variance (2-way ANOVA) or multivariate analysis of variance (MANOVA) followed by Bonferroni *post hoc* test. *p*-values < 0.05 were considered statistically significant.

## Results

### Acute and systemic administration of LPS aggravates long-term memory deficits in 3xTg-AD mice

Approximately 4-month-old (4.27 ± 0.22-month-old) WT and 3xTg-AD mice housed in mild-enriched cages (Figure 1B) were intraperitoneally injected with saline or LPS (1 mg/kg) and spatial memory analyzed using the MWM test after 7 weeks (at the age of 5.89 ± 0.21 months, Figure 1A). During a pilot study we observed that 3xTg-AD mice were able to learn faster when they were pre-exposed to the platform during the handling period and a few minutes before starting the first trial (Figure 1A). Despite of these cautions, 34.6% (*N* = 26) of 3xTg-AD mice did not perform adequately during the MWM trials (Figure 1C) and expressed thigmotactic behavior (see methods) from days 2 or 3. This behavior seemed to be independent of the LPS administration as it was observed in 4 saline and 5 LPS-treated 3xTg-AD mice. Thigmotactic behavior was not observed in WT animals. Therefore, 3xTg-AD mice showing thigmotaxia were excluded from our study.

**Figure 2:**
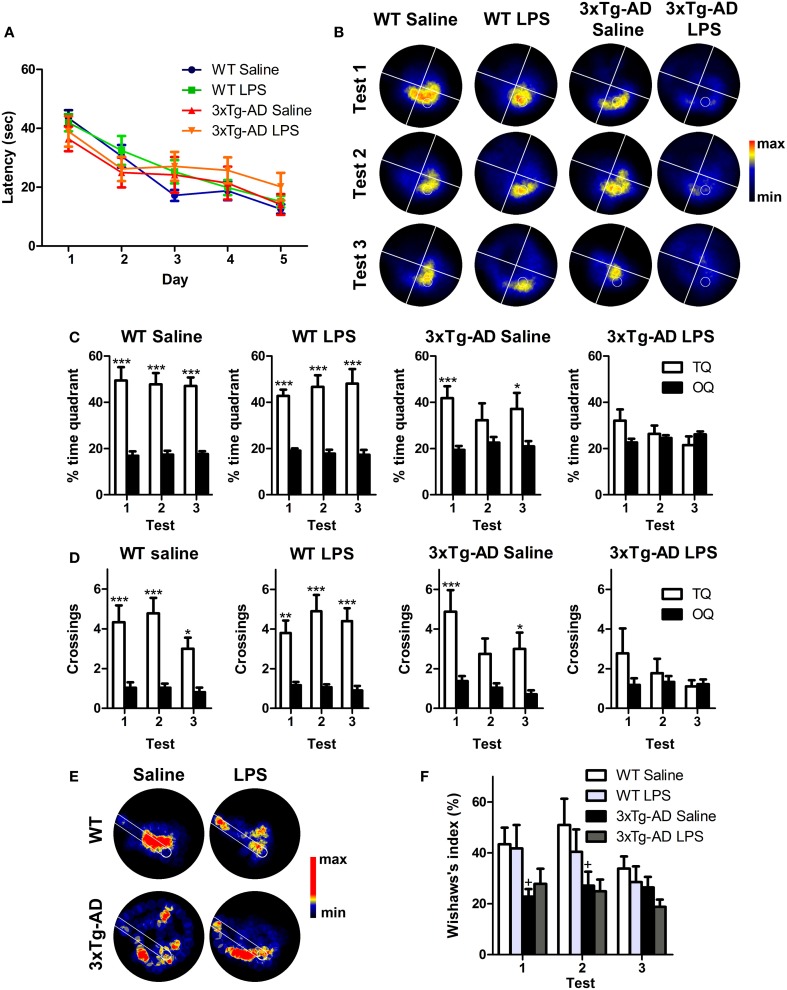
**Long-term detrimental effect of LPS on spatial memory of 3xTg-AD mouse**. **(A)** Performance of WT (*n* = 9), 3xTg-AD (*n* = 8) and LPS-treated WT (*n* = 10) and 3xTg-AD (*n* = 9) mice improved significantly along MWM training session. **(B)** Permanency plots showing the probability of mice location (maximum: red color, minimum: black). White lines divide quadrants and white circle indicates platform location. **(C,D)** Saline and LPS-treated WT mice displayed significantly higher permanencies and crossings in the target quadrant (TQ) compared to the other quadrants (OQ) in all three tests, while 3xTg-AD mice showed such differences only during the first and third trials. 3xTg-AD mice injected with LPS did not show any significant preference for target quadrant. **(E)** Permanency plots before the first crossing to the platform during the first probe test (medium-maximum: red color, minimum: black). White lines delineate a straight corridor from the start position to the platform location. **(F)**. Wishaw's index was significantly different between saline WT and 3xTg-AD mice during tests 1 and 2. Data represent mean ± s.e.m. ^*^p < 0.05, ^**^p < 0.01, ^***^p < 0.001. ^+^*p* < 0.05 (vs. WT mice).

The performance of WT and 3xTg-AD mice improved significantly during the training period reflecting learning (Figure 2A). Statistical analyses showed a significant effect of the interaction day/genotype [multivariate test: *F*_(4, 29)_ = 3.03; *p* < 0.05; within-subjects test: *F*_(4, 128)_ = 3.11; *p* < 0.05] which suggest a different learning pattern between WT and 3xTg-AD mice. However, we did not detect any significant difference between the four groups (WT saline, WT LPS, 3xTg-AD saline, 3xTg-AD LPS) in each particular day [between-subjects effects for genotype: *F*_(1, 32)_ = 0.005; *p* > 0.05]. Three probe tests were performed 24 h after last trial to assess long-term memory. Saline and LPS-treated WT mice showed normal memory function and displayed significantly higher preference to the target quadrant and reached a higher number of crossings to the virtual platform area in all three tests (*p* < 0.001), when compared to virtual platform areas located in non-target quadrants. In 3xTg-AD mice treated with saline these differences were significant only during the first (*p* < 0.001) and third tests (*p* < 0.05) indicating a mild-memory impairment. On the other hand, 3xTg-AD mice injected with LPS did not show any significant preference for target quadrant or platform area (*p* > 0.05, Figures 2B–D), thus showing a great impairment in hippocampal-dependent spatial memory. These results strongly suggest that a single systemic administration of LPS aggravates long-term memory impairment in 3xTg-AD mice.

To evaluate the strategy used by the mice to reach the platform position, we analyzed the incidence of the path traveled within a straight corridor connecting the starting point and the platform area during the probe tests by using the Wishaw's index (Figures 2E,F). Our data revealed that Wishaw's indexes were significantly different between genotypes [between-subjects test: *F*_(1, 32)_ = 12.12; *p* < 0.001]. The results indicate that WT mice use a more direct search strategy to find the platform compared to 3xTg-AD mice. This suggest, that 3xTg-AD mice suffer from impaired hippocampal-dependent memory function (Garthe and Kempermann, 2013).

In summary, our results show that LPS treatment induces a long-term deficit in spatial memory capacities of 6-month-old 3xTg-AD mice (summarized in Figure 7).

### Systemic LPS administration increases the number of new cells incorporated to the dentate gyrus

**Figure 3:**
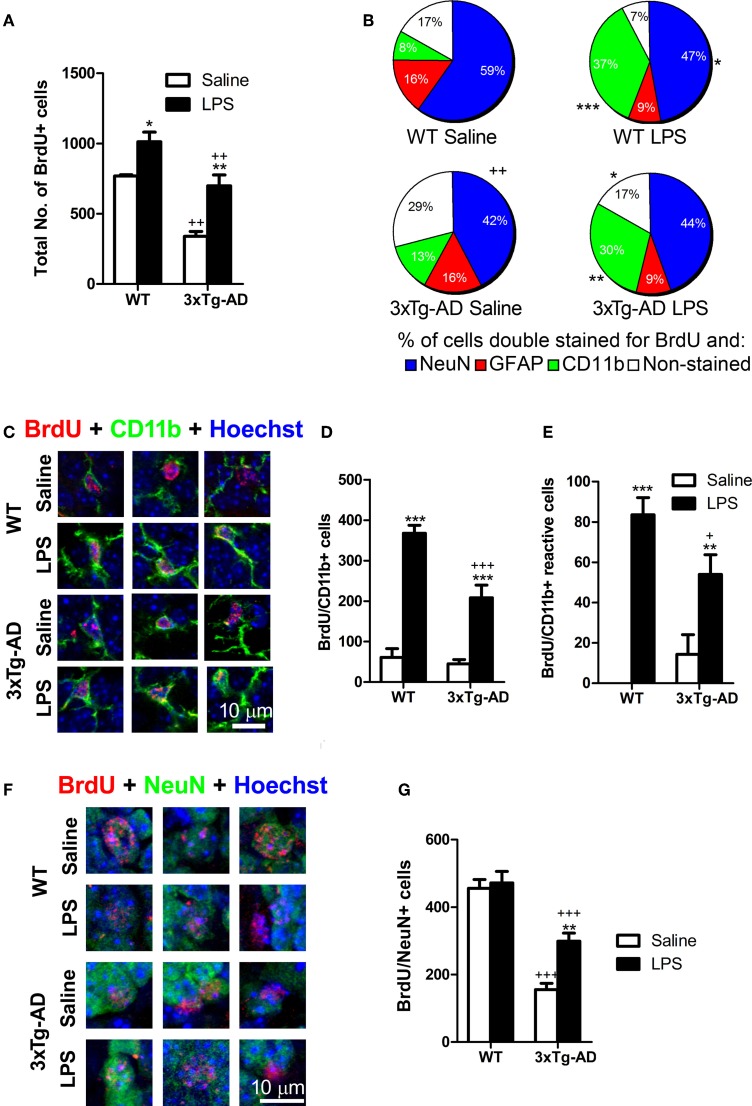
**Net outcome of early LPS effects on different types of proliferating cells**. **(A)** Total number of BrdU cells was significantly reduced in 3xTg-AD mice but increased in the dentate gyrus of mice treated with LPS. **(B)** The proportion of newly-generated neurons (NeuN/BrdU cells) was diminished in 3xTg-AD mice (compared with WT mice) while the percentage of microglial/macrophage cells (CD11b/BrdU+ cells) was increased after LPS treatment. **(C)** Confocal microscopy images showing double stained BrdU/CD11b-positive cells in the granule cell layer/subgranular zone of the dentate gyrus (hypertrophic cells showing thick processes emerging from their cell body were considered as reactive). **(D)** Total number of BrdU-stained microglial/macrophage cells was increased after LPS treatment in both WT and 3xTg-AD mice. However, this increase was smaller in 3xTg-AD mice. **(E)** Total number of reactive BrdU/CD11b-positive cells was increased after LPS treatment. **(F)** Confocal microscopy images showing double stained BrdU/NeuN- positive neurons in the granule cell layer/subgranular zone of the dentate gyrus. **(G)** Total number of BrdU/NeuN-positive neurons was reduced in 3xTg-AD mice but increased by LPS administration. Data represent mean ± s.e.m. ^*^p < 0.05, ^**^p < 0.01, ^***^p < 0.001 (LPS vs. saline-treated mice). ^+^*p* < 0.05, ^++^*p* < 0.01, ^+++^*p* < 0.001 (3xTg-AD vs. WT mice). Number of mice: WT Saline *n* = 4, WT LPS *n* = 5, 3xTg-AD Saline *n* = 5 and 3xTg-AD LPS *n* = 5.

To study whether a single systemic LPS administration changes, at the long-term, the incorporation of new cells to the DG independently of their origin, we labeled cells with the cell proliferation marker BrdU (Figure 1A). Quantitative analysis showed that the number of BrdU-positive cells was reduced (~44%) in the DG of saline-injected 3xTg-AD mice (*p* < 0.01) and increased in both WT (*p* < 0.05; ~32%) and 3xTg-AD (*p* < 0.01; ~106%) after LPS treatment (Figure 3A). As expected, most of the BrdU-positive cells found at the GCL/SGZ of saline-WT mice were relatively mature neurons (NeuN-positive cells, Figure 3B) while just 16% of the BrdU-positive cells expressed GFAP (a marker of astrocytes but also stem cells in the SGZ). The proportion of BrdU/NeuN-positive neurons was statistically lower in 3xTg-AD mice when compared to WT mice (*p* < 0.01, Figure 3B). Interestingly, just a small percentage of BrdU-positive cells (8%) expressed the microglial/macrophage marker CD11b in saline-treated WT mice, indicating that the addition of new microglial/macrophage cells (of non-neural origin) to the DG is relatively low in normal conditions. However, LPS administration increased the proportion of cells double stained for BrdU and CD11b in both WT (*p* < 0.001) and 3xTg-AD (*p* < 0.01) mice. In agreement, statistical analysis revealed an increase in the total number of BrdU/CD11b-positive cells after LPS administration in WT and 3xTg-AD mice (Figures 3C,D). We did not find any BrdU/CD11b-positive cell with a clear reactive morphology in saline-treated WT mice and just few were found in saline-3xTg-AD mice [no significant effect of genotype: *F*_(1, 15)_ = 0.78; *p* > 0.05, Figures 3C,E]. However, LPS-treated mice, both WT and 3xTg-AD, exhibited an increase in the number of newly-generated microglia/macrophage cells with reactive-like morphology (BrdU/CD11b, Figures 3C,E). Interestingly, the increase in the number of newly-generated microglia/macrophage cells induced by LPS was significantly smaller in 3xTg-AD mice [6-fold and 4.6-fold increase in WT and 3xTg-AD mice, respectively; *p* < 0.001; genotype and treatment interaction effect: *F*_(1, 15)_ = 8.102; *p* < 0.05 Figure 3D], and something similar occurred with reactive-like CD11b/BrdU-positive cells [*p* < 0.05, genotype and treatment interaction effect: *F*_(1, 15)_ = 6.51; *p* < 0.05, Figure 3E]. These results reveal that 3xTg-AD mice have a reduced capacity of microglial response after LPS challenge when compared to WT mice. Moreover, we also quantified the proportion of GCL area occupied by CD11b staining as a general measurement of microglial activation and no significant differences were found between the 4 groups analyzed (data not shown).

These findings clearly demonstrate that a single and systemic LPS administration increases the production of new cells in the DG of mice by augmenting, mainly, the proportion of newly-generated microglial/macrophage cells as part of the neuroinflammatory process.

### Changes in the population of new neurons derived from precursor cells influenced by early LPS effects

Previous studies have demonstrated that intraperitoneal administration of LPS in rodents induces an early decrease in the number of DG proliferating cells (Bastos et al., 2008; Bachstetter et al., 2010; Fujioka and Akema, 2010; Sierra et al., 2010). However, none of these reports analyzed the net impact of this early LPS effect in the final number of DG newly-generated neurons in WT or AD mouse models. For such purpose, mice were injected with BrdU twice a day, 2 days before and after LPS administration, and sacrificed ~7 weeks later (see discussion). As expected, we detected a 2.9-fold reduction in the number of newly-generated neurons (NeuN/BrdU; *post hoc* test: *p* < 0.001) in the DG of saline administered 3xTg-AD mice when compared to WT mice (Figures 3F,G). Surprisingly, LPS administration induced a 1.92-fold increase in the number of NeuN/BrdU stained cells exclusively in 3xTg-AD mice (saline vs. LPS 3xTg-AD mice: *p* < 0.01) while it had no significant effect on WT mice (saline vs. LPS WT mice: *p* > 0.05). However, despite of the increase induced by LPS, the number of newly-generated NeuN/BrdU-positive neurons in 3xTg-AD was still significantly reduced (1.58 fold decrease) when compared to WT mice (LPS 3xTg-AD mice vs. LPS WT mice: *p* < 0.001). These results indicate that, in 3xTg-AD mice, initial effects of LPS administration induce a moderated recovery in the incorporation of new neurons to the DG.

### Systemic LPS administration reduces the number of doublecortin-positive cells and their size in WT mice

**Figure 4:**
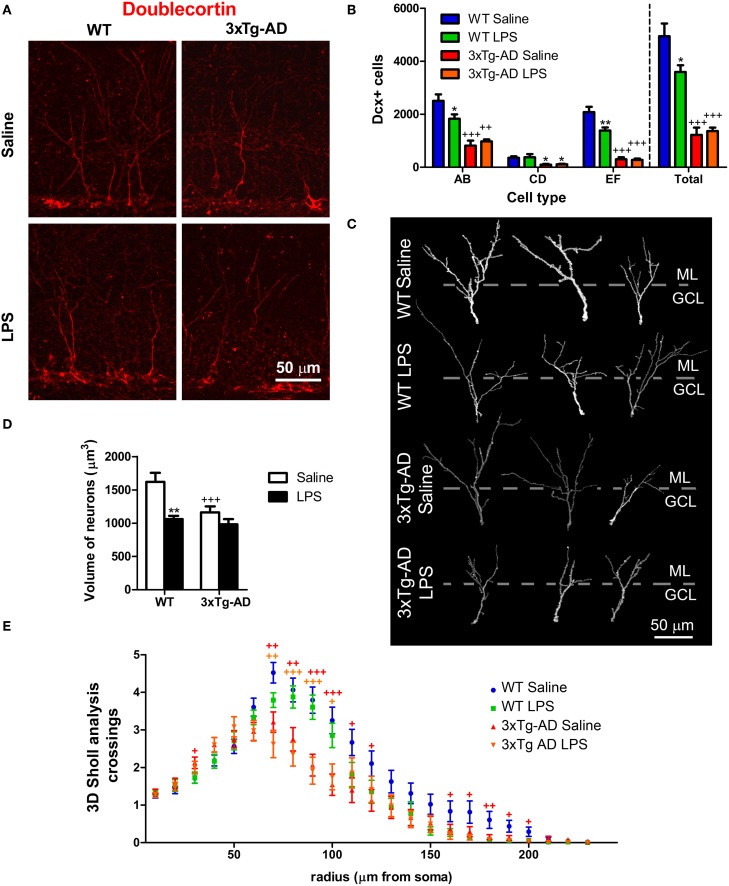
**LPS effect on number and morphology of doublecortin-positive cells**. **(A)** Representative confocal microscopy images showing Dcx-positive cells in the dentate gyrus (DG). **(B)** Quantitative analysis reveals a significant reduction in the number of Dcx-labeled cells in the DG of 3xTg-AD mice compared with WT mice. The number of AB and EF-type cells, but not CD-type, was significantly decreased in WT mice ~7 weeks after LPS treatment. **(C)** Three dimensional reconstructions of representative doublecortin (Dcx) stained cells of the DG. Dotted lines separate molecular (ML) from granular cell layer (GCL). **(D)** The volume of the dendritic tree of Dcx-positive cells was reduced in 3xTg-AD mice and in LPS-treated WT mice. **(E)** 3D Sholl analysis revealed reduced number of dendritic intersections with Sholl spheres in radius 70–120 and 160–200 μm in 3xTg-AD mice. ^*^p < 0.05, ^**^p < 0.01 (LPS vs. saline-treated mice). ^+^*p* < 0.05, ^++^*p* < 0.01, ^+++^*p* < 0.001 (vs. WT mice). Number of mice: WT Saline *n* = 4, WT LPS *n* = 5, 3xTg-AD Saline *n* = 5 and 3xTg-AD LPS *n* = 5.

To further study the long-term effect of LPS on a younger population of newborn neurons originated long (at least 3 weeks) after LPS administration, we analyzed the number of cells stained for Dcx. Dcx is a cytoskeletal-related molecule expressed in the DG by cells ranging from early neuroblasts to 28 days old neurons (Rao and Shetty, 2004; Karten et al., 2006; Plümpe et al., 2006). We observed a 4.05-fold decrease in the total number of Dcx-positive cells in the GCL/SGZ of 3xTg-AD mice [Figures 4A,B, overall genotype effect: *F*_(1, 16)_ = 112.65; *p* < 0.001; *post hoc* test WT saline vs. 3xTg-AD saline mice: *p* < 0.001]. Dcx is present in a heterogeneous population of neuronal cells that has been previously categorized in six different types according to their morphological maturation stage: from type A to F (Plümpe et al., 2006). Therefore, we analyzed the number and proportion of AB (proliferative), CD (intermediate) and EF (postmitotic and almost mature) Dcx-positive cells (Figure 4B) as previously described (Valero et al., 2011). All different cell populations were reduced in 3xTg-AD mice compared to WT mice (−3.07-fold for AB cells; *p* < 0.001; −3.58-fold for CD cells; *p* < 0.05; −6.86-fold for EF cells; *p* < 0.001). Moreover, LPS treatment reduced the total number of DG Dcx-positive cells (−1.52-fold *p* < 0.01), specifically affecting AB and EF cells in WT mice (−1.37-fold for AB cells; *p* < 0.05; −1.50-fold for EF cells; *p* < 0.001), but did not cause any significant change in 3xTg-AD mice (Figure 4B).

In a previous study we observed a reduction in the dendritic complexity and size of Dcx-positive cells in the APP_Sw,Ind_ mouse model of AD (Valero et al., 2011). In agreement, our data revealed a ~28% volumetric reduction in the dendritic tree of EF cells from 3xTg-AD mice (vs. WT saline, *p* < 0.001, Figures 4C,D). Moreover, EF Dcx-positive cells from WT mice treated with LPS showed a ~36% reduction in their volume (vs. WT-saline, *p* < 0.001). Interestingly, further volumetric analysis revealed that LPS treatment induced a decrease in the proportion of the dendritic tree located at the outer/mid ML (the region of contact with input fibers arriving from the entorhinal cortex, Amaral et al., 2007) in WT mice (*p* < 0.01). Furthermore, we also examined the dendritic morphology of Dcx-positive cells using 3D Sholl analysis (Figure 4E). Sholl analysis revealed an overall effect of genotype [Multivariate Test: *F*_(21, 168)_ = 2.53; *p* < 0.001] but no significant effect of LPS treatment [Multivariate Test: *F*_(21, 168)_ = 0.79; *p* > 0.05] suggesting a different branching pattern between Dcx-positive neurons of WT and 3xTg-AD mice. We found a significant increase in the number of intersections in 3xTg-AD mice at the GCL (radius 30) and a decrease in the ML from 70 to 120 μm radius and from 160 to 200 μm radius (Figure 4E).

In summary, our results show new evidences about the profound impairment of adult neurogenesis occurring in 3xTg-AD mice but also demonstrate that LPS treatment had a specific long-term effect in Dcx-positive cells from WT mice, reducing their number and their size (Figure 7).

### LPS treatment decreases the number of synaptic puncta in the dendrites of doublecortin-positive cells

**Figure 5:**
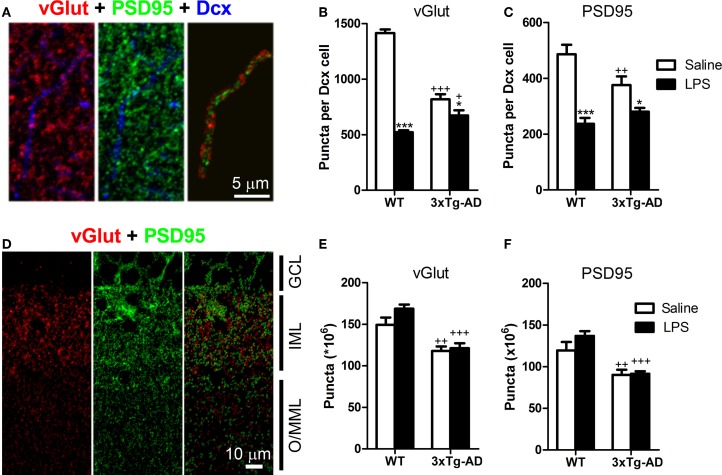
**LPS treatment decreases the number of synaptic puncta in the dendrites of doublecortin-positive cells**. **(A)** Confocal microscope image showing a doublecortin (Dcx)-positive dendrite in the outer/mid molecular layer (O/MML) and colocalizing PSD95-positive post-synaptic or juxtaposed vGlut-positive presynaptic puncta. **(B,C)** 3xTg-AD showed a significant decrease in the number of O/MML vGlut and PSD95-positive puncta per Dcx-positive cell. LPS reduced synaptic puncta in WT and 3xTg-AD Dcx-positive cells. **(D)** Confocal microscope image showing vGlut and PSD95 staining in the granular cell layer (GCL), inner molecular layer (IML), and O/MML. Two-color images show segmented vGlut (red) and PSD95 (green)-positive puncta (see methods) for automatic quantification. **(E,F)** A significant decrease in the number of vGlut and PSD95-positive puncta was found in the O/MML of 3xTg-AD mice with no effects of LPS treatment. ^*^p < 0.05, ^***^p < 0.001 (LPS vs. saline-treated mice). ^+^*p* < 0.05, ^++^*p* < 0.01, ^+++^*p* < 0.001 (vs. WT mice). Number of mice: WT Saline *n* = 4, WT LPS *n* = 5, 3xTg-AD Saline *n* = 5 and 3xTg-AD LPS *n* = 5.

We analyzed the number of postsynaptic (PSD95-positive) and presynaptic (vGlut-positive) puncta and their colocalization/juxtaposition with Dcx-positive fibers in the outer/mid ML of the DG (Figures 5A–C). Our analysis showed a significant reduction in the number of outer/mid ML vGlut (−1.73-fold) and PSD95-positive (−1.37-fold) puncta of Dcx-positive cells of 3xTg-AD mice (Figures 5B,C). Furthermore, LPS treatment decreased the total number of outer/mid ML vGlut [*F*_(1, 16)_ = 172.20; *p* < 0.001] and PSD95-positive puncta per Dcx cell in both WT and 3xTg-AD mice [*F*_(1, 16)_ = 41.52; *p* < 0.001]. However, LPS effect was stronger in WT mice (fold changes for vGlut: −2.71, *p* < 0.001; PSD95: −2.05, *p* < 0.001) than in 3xTg-AD mice (fold changes for vGlut: −1.22, *p* < 0.05; PSD95: −1.26, *p* < 0.05). Interestingly, we detected a 1.33-fold increase in the volumetric density of PSD95 puncta per Dcx-positive fiber in WT mice treated with LPS (*p* < 0.01). This increase in post-synaptic puncta could constitute a failed attempt to compensate the reduction of dendritic size induced by LPS in WT mice.

Importantly, LPS treatment did not produce a generalized decrease of synaptic puncta at the outer/mid ML, as no effects of LPS administration were observed in the total number of vGlut and PSD95-positive puncta (Figures 5D–F). However, vGlut and PSD95-positive puncta were clearly reduced in 3xTg-AD mice when compared with WT mice (fold decrease for vGlut: −1.33, *p* < 0.001; PSD95: −1.41, *p* < 0.001).

We also analyzed pre- and post-synaptic puncta at the inner ML and observed an overall decrease in the number of vGlut [*F*_(1, 16)_ = 180.00, *p* < 0.001; −1.98-fold decrease] and PSD95-positive puncta [*F*_(1, 16)_ = 44.13, *p* < 0.001; −1.63-fold decrease] in 3xTg-AD mice. On this layer, LPS injection also induced a decrease in the number of vGlut [*F*_(1, 16)_ = 66.63, *p* < 0.001; −1.56-fold] and PSD95-positive puncta [*F*_(1, 16)_ = 12.53, *p* < 0.01; −1.31-fold] of Dcx cells from WT mice but no effect was detected in 3xTg-AD mice.

These data indicate that LPS treatment impairs the formation of synaptic specializations in the dendrites of Dcx-positive cells in the outer/mid ML of both WT and 3xTg-mice. This may have important consequences at the functional level considering that the contact between axons from the entorhinal cortex and dendrites of DG granule cells mainly occurs at the outer/mid ML (Amaral et al., 2007).

### LPS induces a mild impairment of spatial memory in wild type mice

**Figure 6:**
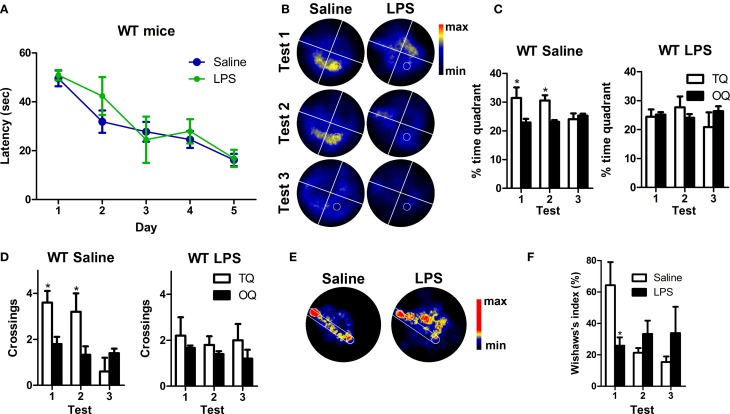
**LPS treatment induces mild impairment of spatial memory in WT mice**. **(A)** The performance of saline (*n* = 5) and LPS-treated WT (*n* = 5) mice improved significantly along MWM training sessions. **(B)** Permanency plots showing the probability of mice location (maximum: red color, minimum: black). White lines divide quadrants and white circle indicates platform location. **(C,D)** Saline WT mice displayed significantly higher permanencies and crossings in the target quadrant (TQ) compared to the other quadrants (OQ) during the first and second tests. LPS-treated mice did not show any significant preference for target quadrant or platform crossings. **(E)** Permanency plots before the first crossing to the platform during the first probe test (medium-maximum: red color, minimum: black). White lines delineate a straight corridor from the start position to the platform location. **(F)** Data revealed a significant difference in Wishaw's index between saline and LPS-treated mice during the first probe test. Data represent mean ± s.e.m. ^*^p < 0.05.

**Figure 7:**
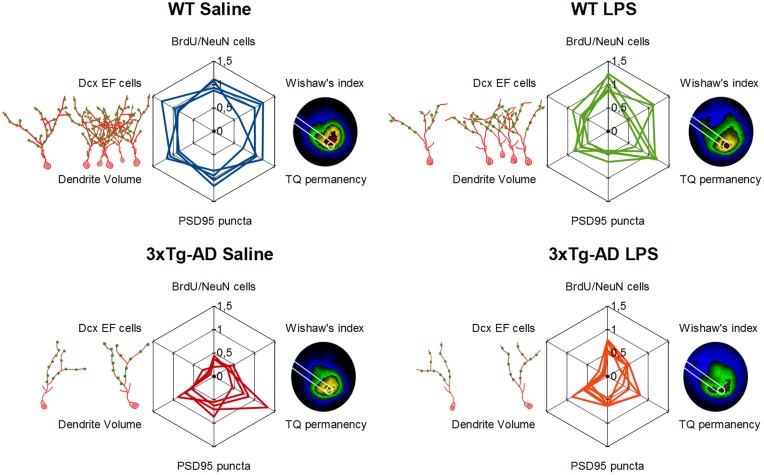
**Detrimental long-term effect of LPS induced inflammation in hippocampal neurogenic reserve and memory function**. Schematic representation of main results. Doublecortin-positive EF cells, their morphology (red drawing) and synaptic puncta (green dots) are represented on the left of each web graphs while a 5-colored version of permanency plots appears on their right, different areas are colored depending on permanency (from high to lower permanency levels colors are: red, yellow, green, blue, and black). Web graphs are referred to mean values of WT-Saline mice.

Taken together, our data clearly show a decrease in the number of Dcx-positive cells, their volume and their number of synaptic puncta in WT mice after LPS treatment (Figure 7). However, despite of the big impact that LPS injection has on Dcx-positive cells we did not detect any effect using our initial version of MWM protocol that was designed to facilitate learning in 3xTg-AD mice. Therefore, we performed a standard MWM test with saline or LPS injected WT mice in which animals were not pre-exposed to the platform. Repeated measures test revealed a significant reduction in the latency to reach the platform along the trials [multivariate test: *F*_(4, 5)_ = 44.38, *p* < 0.001] and no differences due to LPS treatment (Figure 6A). Thus, as previously observed, LPS administration did not affect learning. However, contrarily to WT mice treated with saline, WT mice treated with LPS did not show any significant preference for the target quadrant (Figures 6B,C) or area of the virtual platform (*p* > 0.05, Figure 6D), indicating no spatial memory retention. Furthermore, our data revealed a significant decrease in Wishaw's index in LPS WT mice during test 1 (*p* < 0.05, Figures 6E,F), indicating the use of a less direct path to reach the platform.

These data demonstrate that long after systemic inflammation induction, spatial memory is impaired in WT mice, giving functional support to the observed effect of LPS in the number, morphology and synaptic puncta of Dcx-positive newly-generated neurons.

## Discussion

Systemic inflammation and concomitant neuroinflammation have been suggested to trigger cognitive decline, neurodegeneration and also AD pathology (Qin et al., 2007; Herrup, 2010; Terrando et al., 2011, 2013). In this study, we have evaluated spatial memory and adult neurogenesis in WT and 3xTg-AD mice 7 weeks after the induction of systemic inflammation by intraperitoneal administration of LPS. We used ~6 months-old 3xTg-AD mice, at this age 3xTg-AD mice are characterized by increased levels of intracellular Aβ peptide in the hippocampus, but absence of amyloid plaques or hyperphosphorylated tau protein (Oddo, 2003; Billings et al., 2005), while we detected relatively mild memory deficits and a profound impairment of adult neurogenesis on these mice. Furthermore, we demonstrate here that a single systemic administration of LPS have an important long-term impact in the DG of both WT and 3xTg-AD mice, reducing fundamental elements of the neurogenic reserve and concomitantly impairing spatial memory (main results summarized in Figure 7).

It has been previously described that an acute administration of LPS, similar to that used here, reduces DG adult neurogenesis at the short-term. However, there is some controversy about whether this effect is due to a decrease in cell proliferation (Bachstetter et al., 2010; Fujioka and Akema, 2010) or to an increase in cell death (Bastos et al., 2008; Sierra et al., 2010). Apparent contradictions between these studies may be related to the different timings of deoxythymidine analog administration. Thus, each one of the above mentioned studies targeted a different population of new cells affected by LPS at one specific and unique maturational stage (proliferating, survival or final differentiation phases). Our BrdU administration protocol (Figure 1A) was aimed to analyze long-term consequences of LPS treatment to the net incorporation of new neurons, considering that this net effect is the most relevant for DG function. Therefore, we tagged with BrdU a relatively broad range of dividing-progenitors to study cells affected by LPS administration in either proliferating or survival phases. As far as we know, there is just another study analyzing long-term effects of acute LPS administration in memory and adult neurogenesis in WT mice (Ormerod et al., 2013) but not in an AD mouse model. Surprisingly, our results showed that the net number of BrdU-positive newborn neurons was not influenced by LPS administration in WT mice while it was augmented in 3xTg-AD mice. A similar increase in BrdU/NeuN-positive cells (28 days after BrdU and LPS administration) has been previously described in adult rats treated with a high dose of LPS (Bland et al., 2010). The most plausible explanation for this phenomenon is that the initial reduction of neuronal progenitors induced by LPS was compensated (or overcompensated in 3xTg-AD mice) by an increase in cell proliferation or/and cell survival of the remaining cells. However, our data and those from Ormerod et al. (2013) point to the existence of more profound and long-lasting detrimental effects of systemic inflammation on memory and adult neurogenesis than initially thought. The time extend of such effects on cognition should be analyzed in future works. Nevertheless, the decrease in mitotic neuroblasts (AB cells) of LPS-treated WT mice (Figure 4B) suggests that the effects of systemic inflammation in adult neurogenesis may be prolonged further over time.

In agreement with previous publications indicating that LPS-induced neuroinflammation can lead to AD-like pathology in rodents (Hauss-Wegrzyniak et al., 1998; Lee et al., 2008; Choi et al., 2012; Kahn et al., 2012), memory and neurogenic changes observed in LPS-treated WT mice resemble a mild-version of the deficiencies shown by saline-treated 3xTg-AD mice (Figure 7). Interestingly, many of the parameters changed long after LPS injection in WT mice remained unaltered in LPS-treated 3xTg-AD mice, except for the number of synaptic puncta that was decreased in both groups. Several evidences indicate that the population of 1–4 weeks old immature Dcx-positive neurons is crucial for hippocampal memory function (Deng et al., 2009; Trouche et al., 2009; Piatti et al., 2011; Spampanato et al., 2012). Thus, the reduction in the number of synaptic puncta, summed to the pre-existent (in the case of 3xTg-AD mice) or concomitant (in the case of LPS-injected WT mice) decrease in number and size of DG new-neurons, may trigger the impairment of spatial memory function observed long time after LPS administration (notice the variation in the vertex corresponding to synaptic puncta in web graphs for LPS-treated mice of Figure 7). Some of the neurogenic and memory deficits described here in 3xTg-AD and LPS-injected WT mice were previously observed in another mouse model of AD (APP_Swe, Ind_ mice) and overcame after being exposed to environmental enrichment (Valero et al., 2011). However, in the present study we used a mild-enriched environment (Figure 1B) that was unable to compensate the detrimental action of LPS. Thus, future work should address whether environmental enrichment is able to avoid negative effects mediated by LPS.

Our study confirmed that systemic inflammation triggers long-lasting memory deficits, worsening AD-related impairment in memory and adult neurogenesis. Based on our data, we propose that depending on the state of the cognitive reserve, the effect of a single peripheral inflammatory event can lead from almost inappreciable cognitive changes to the manifestation of AD related pathological deficits. Therefore, knowing the most effective way to avoid or compensate brain cognitive reserve deterioration should be a priority to prevent cognitive decline during ageing and after exposure to inflammatory events.

## Author contributions

Conceived and designed the experiments: Jorge Valero and João O. Malva performed the experiments: Jorge Valero, Ismael Neiva, Giorgia Mastrella, and João O. Malva analyzed the data: Silvia Sánchez and Jorge Valero prepared figures: Silvia Sánchez and Jorge Valero wrote the paper: Jorge Valero and João O. Malva.

### Conflict of interest statement

The authors declare that the research was conducted in the absence of any commercial or financial relationships that could be construed as a potential conflict of interest.
